# Prevalence of Hepatitis C virus genotypes in nine selected European countries: A systematic review

**DOI:** 10.1002/jcla.22876

**Published:** 2019-03-07

**Authors:** Arnolfo Petruzziello, Giovanna Loquercio, Rocco Sabatino, Daniel Vasile Balaban, Najeeb Ullah Khan, Mauro Piccirillo, Luis Rodrigo, Lucia di Capua, Annunziata Guzzo, Francesco Labonia, Gerardo Botti

**Affiliations:** ^1^ UOC Clinical Pathology AORN Sant’Anna e San Sebastiano Caserta Italy; ^2^ SSD Virology and Molecular Biology, Department of Diagnostic Area Istituto Nazionale Tumori – Fondazione “G. Pascale”, IRCCS Italia Naples Italy; ^3^ Carol Davila" University of Medicine and Pharmacy, "Dr. Carol Davila" Central Military Emergency University Hospital Bucharest Romania; ^4^ Institute of Biotechnology and Genetic Engineering (Health Davison) The University of Agriculture Peshawar Pakistan; ^5^ Hepatobiliar and Pancreatic Unit, Department of Surgical Oncology Istituto Nazionale Tumori–Fondazione “G. Pascale” IRCCS Italia Naples Italy; ^6^ Gastroenterology Service Hospital Universitario Central de Asturias, University of Oviedo Oviedo Spain; ^7^ Scientific Director IRCCS Fondazione Pascale Naples Italy

**Keywords:** epidemiology, HCV genotype, HCV infection, HCV prevalence, Hepatitis C virus

## Abstract

**Background:**

Hepatitis C virus (HCV) infection is a global health problem especially for its increasing level of mortality. Detailed knowledge of HCV genotypes prevalence has clinical relevance since the efficacy of therapies is impacted by genotypes and subtypes distribution. Moreover, HCV exhibits a great genetic variability regionally.

To date, there are no published studies assessing HCV genotypes distribution in specific countries of the Mediterranean basin. The aim of this study was to review data published from 2000 to 2017 with the purpose to estimate genotypes distribution of HCV infection in nine European countries all located in the Mediterranean basin.

**Methods:**

A systematic research of peer‐reviewed journals indexed in PubMed, Scopus, and EMBASE databases selected if containing data regarding distribution of HCV genotypes in nine selected European countries (Albania, Bosnia, Croatia, France, Greece, Italy, Montenegro, Slovenia, and Spain) was performed.

**Results:**

Genotype 1 is the most common (61.0%), ranging from 80.0% in Croatia to 46.0% in Greece, followed by genotype 3 (20.0%), varying from 38.0% in Slovenia to 7.0% and 8.0%, respectively, in Italy and in Albania and by genotype 4 (10.0%) that shows an increase of 1.1% with respect to data obtained till 2014 probably due to the increasing migrants arrivals to Southern Europe. G2, the fourth most frequent genotype (8.5%), particularly common in Italy (27.0%) and Albania (18.0%) might be probably introduced in Southern Italy as a result of Albanian campaign during Second World War and more and more increased by the migration flows from Albania to Italy in the 90s.

**Conclusion:**

Epidemiology of HCV infection shows a high variability across the European countries that border the Mediterranean Sea. HCV genotyping is a relevant tool to monitor the dynamic process influenced by both evolving transmission trends and new migration flows on HCV scenario.

## 1 INTRODUCTION

Hepatitis C virus (HCV) with about 3‐4 million people infected every year and over 350 000 deaths is one of the main leading cause of liver‐related death worldwide.^1^, ^2^, ^3^, ^4^ It has been estimated that over 71 million people have chronic hepatitis C infection mainly among populations of WHO Eastern Mediterranean and European Regions.^3^, ^5^ Its high mortality rate seems to be essentially due to the fact that persistent HCV infection is often associated with the development of liver cirrhosis and hepatocellular carcinoma (HCC).^6^, ^7^, ^8^, ^9^, ^10^, ^11^

Seven HCV genotypes have been up to now identified, each comprising multiple subtypes (1a, 1b, and so on) differing from each other by 31%‐33% over the whole viral genome.^12^, ^13^, ^14^, ^15^ This high genetic diversity poses an obstacle not only for vaccine development but also for an effective antiviral therapy since its duration and response rate may be greatly influenced by the different isolated viral strains.

Detailed knowledge of HCV genotype has a great clinical relevance since the efficacy of therapies measured through the rate of sustained virological response (SVR), defined as the rate of persistent viremia 24 weeks after the end of antiviral therapy, is greatly impacted by genotypes and subtypes distribution.^16^

Previously, the standard therapy for the treatment of HCV infections was based on PEG‐IFNα/RBV,^16^, ^17^, ^18^, ^19^, ^20^ that if compared to other antiviral drugs, seemed to positively influence the rates of SVR.^21^ However, it has been widely described that the IFNα/RBV treatment may cause many adverse events (ie, poor tolerability, suboptimal efficacy, and prolonged treatment course) unlike new and expensive direct‐acting antivirals DAAs,^21^ introduced recently. DAAs, which specifically inhibit viral proteins essential for viral replication, seem to improve the rates of SVR.^22^

Thus, it is clear that a better survey of HCV epidemiology, especially focused on the knowledge of different genotypes distribution worldwide, could essentially help to reduce the effects of this severe pandemic disease.^6^, ^23^

The geographic distribution of HCV genotypes is heterogeneous and characterized by a distribution of the “epidemic subtypes” (1a, 1b, 2a, and 3a) in high‐income countries and of the “endemic” strains in restricted areas, as West Africa, Southern Asia, Central Africa, and Southeastern Asia.^5^, ^24^

Regarding Europe, as previously reported, the Global Burden Diseases project subdivides it into three main areas: Central, Eastern, and Western.^25^, ^26^, ^27^ As previously described, genotype distribution does not show high variability among the three macro‐areas: genotype 1 (G1) seems to be equally distributed among them (70.0% in Central Europe, 68.1% in Eastern Europe, and 55.1% in Western Europe), like genotype 3 (G3) (29.0% in Western Europe, 26.6% in Eastern Europe, and 21.0% in Central Europe). Genotype 2 (G2), instead, seems to be most common in Western Europe (8.9%), while genotype 4 (G4) is essentially present in Central and Western Europe (4.9% and 5.8%, respectively).^24^, ^25^, ^26^, ^27^, ^28^

The European Mediterranean basin is a particularly interesting area regarding this context not only because its shores are close to different geographical areas (Northern Africa and Middle Eastern), each characterized by a greatly different HCV epidemiology and healthcare systems, but especially for the influence thatmigration flows might have had in the last decades.^24^ It may be suggestive the hypothesis that the epidemiological status of HCV infection in this area had been radically changed in the past, considering the colonial status of the majority part of the Northern Africa in the first half of ninth century and the increasing numbers of migrants moved from Africa and Middle Eastern to Southern Europe in the last two decades.

Even if it is well known that epidemiological characteristics of HCV infection in this area differ significantly country by country considering the different historical context, social status, evolution of the different healthcare systems, and many other reasons, it is undeniable that the knowledge of the epidemiological characteristics of HCV infection in this area could give a rational background for better understand future evolution of the infection in an area that it is considered the door to Europe. In this context, HCV genotyping could be an important epidemiological tool to monitor the effect of migration flows.

The aim of this study has been to improve our knowledge about HCV genotypes distribution through an analysis of data published from 2000 to 2017 in nine European countries all located in the Mediterranean basin.

## 2 MATERIALS AND METHODS

We conducted a systematic research of peer‐reviewed journals indexed in PubMed, Scopus and EMBASE databases selected if containing data regarding distribution of HCV genotypes in nine selected European countries. Among the WHO European countries, we considered only those that geographically shore the Mediterranean Sea, specifically Albania, Bosnia, Croatia, France, Greece, Italy, Montenegro, Slovenia, and Spain. Malta and Cyprus were excluded since no updated data were available.

References were identified through indexed articles found by searching in the abovementioned databases using the following terms: ‘‘[Country Name] and [hepatitis C or HCV] and [genotypes].’’ Articles were scored based on the study sample size (>200 subjects) and the age of the studied population (>20 years old). Studies in nonrepresentative populations (eg, people who inject drugs ‐PWID's‐, hemophiliacs, blood donors, etc) were excluded.

In order to assure a formal evaluation of the methodological quality of the included studies, we decided to use as selection method the PRISMA system (preferred reporting items for systematic reviews and meta‐analyses).^29^

The PRISMA statement is an evolution of the original QUOROM guideline and consists of a 27‐item checklist and a four‐phase flow diagram. The checklist includes items deemed essential for the evaluation of a systematic review and referred to every section of the article (title, introduction, methods, and so on). Following this methodology, articles were selected only if responsive of the following inclusion criteria: (a) studies in which the sample population was enrolled from one of the selected countries between January 2000 up to December 2017; (b) English full‐text articles concerning the HCV genotypes prevalence from the nine selected countries; and (c) studies considering only HCV‐RNA‐positive patients in which genotyping has been performed through a standard genotyping method. Sequencing and phylogenetic analysis of the core/E1 or NS5B region are nowadays considered to be the gold standard for HCV genotyping since it allows to accurately identify the subtype,^30^, ^31^ we chose only articles in which these methods were used. The currently available commercial techniques for HCV subtyping fail in 2%‐10% of samples because of inaccurate genotyping, failure to amplify or to categorize. The majority of commercial assays are based on the amplification of short HCV RNA regions from clinical specimens, followed by a type‐specific assay, such as line probe reverse hybridization,^32^, ^33^ or sequence analysis.^34^, ^35^

On the contrary, we excluded the following articles: (a) studies with lacking data (age, size, or sex of the samples); (b) studies concerning only serologic data; and (c) studies not included to the inclusion criteria. Selected articles were then reviewed by four independent reviewers (BDV, KNU, PM, and RL), and in case of uncertainty, the article was analyzed by the supervisor of the study (PA). Since the majority of selected articles described HCV cases at the genotype level, we preferred to use this classification as proposed by Simmonds et al.^14^. Moreover, we decided to report only data regarding the most common genotypes in the studied area (genotypes 1, 2, 3, 4), while less common ones, as genotypes 5 or 6, or mixed infections were classified as “others.” Genotype 7 was not included in the analysis since we did not found any article reporting this genotype in the selected area.

HCV genotypes distribution was investigated through Stata software version 14 (Stata Corp, College Station, TX, USA), and statistical heterogeneity was explored using the I‐square at the 5% significance level. Pooled mean proportions were estimated for each genotype and by country using DerSimonian‐Laird random‐effects meta‐analyses.

## 3 RESULTS

**Figure 1:**
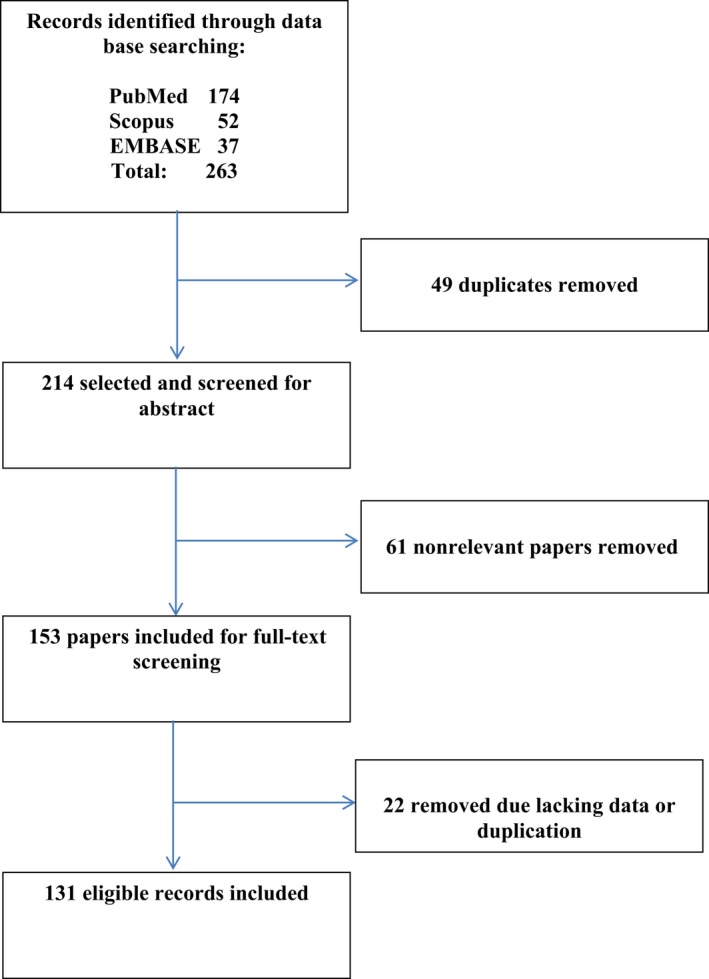
Flowchart of systematic literature search and article selection (PRISMA guideline 2009)

Applying our inclusion criteria, we initially identified 263 articles and then reduced to 214 after exclusion of 49 duplicates and to 131 eliminating articles that reported not complete data (Figure 1).

Globally, we considered 43 studies from Italy (23 041 subjects), 36 from Spain (19 407 subjects), 32 from France (13 270 subjects), 7 from Greece (2505 subjects), and 6 from Croatia (3541 subjects). Only three studies were selected from Slovenia (795 subjects) and 2 from Montenegro (401 patients) and only single studies from Albania and Bosnia. A description of the populations enrolled in the studies is summarized in Table 1.

**Table 1:** Populations enrolled in the selected studies

Countries	Studies (n)	Subjects (n)	General population (n)	HCV‐positive outpatients (n)	Chronic HCV patients (n)	Mixed populations (n)
Albania	1	50	1	0	0	0
Bosnia	1	75	0	1	0	0
Croazia	6	3541	2	2	1	0
Francia	32	13 270	14	10	6	2
Grecia	7	2505	3	3	1	0
Italia	43	23 041	18	9	12	4
Montenegro	2	401	1	1	0	0
Slovenia	3	795	2	1	0	0
Spain	36	19 407	18	10	6	2

**Figure 2:**
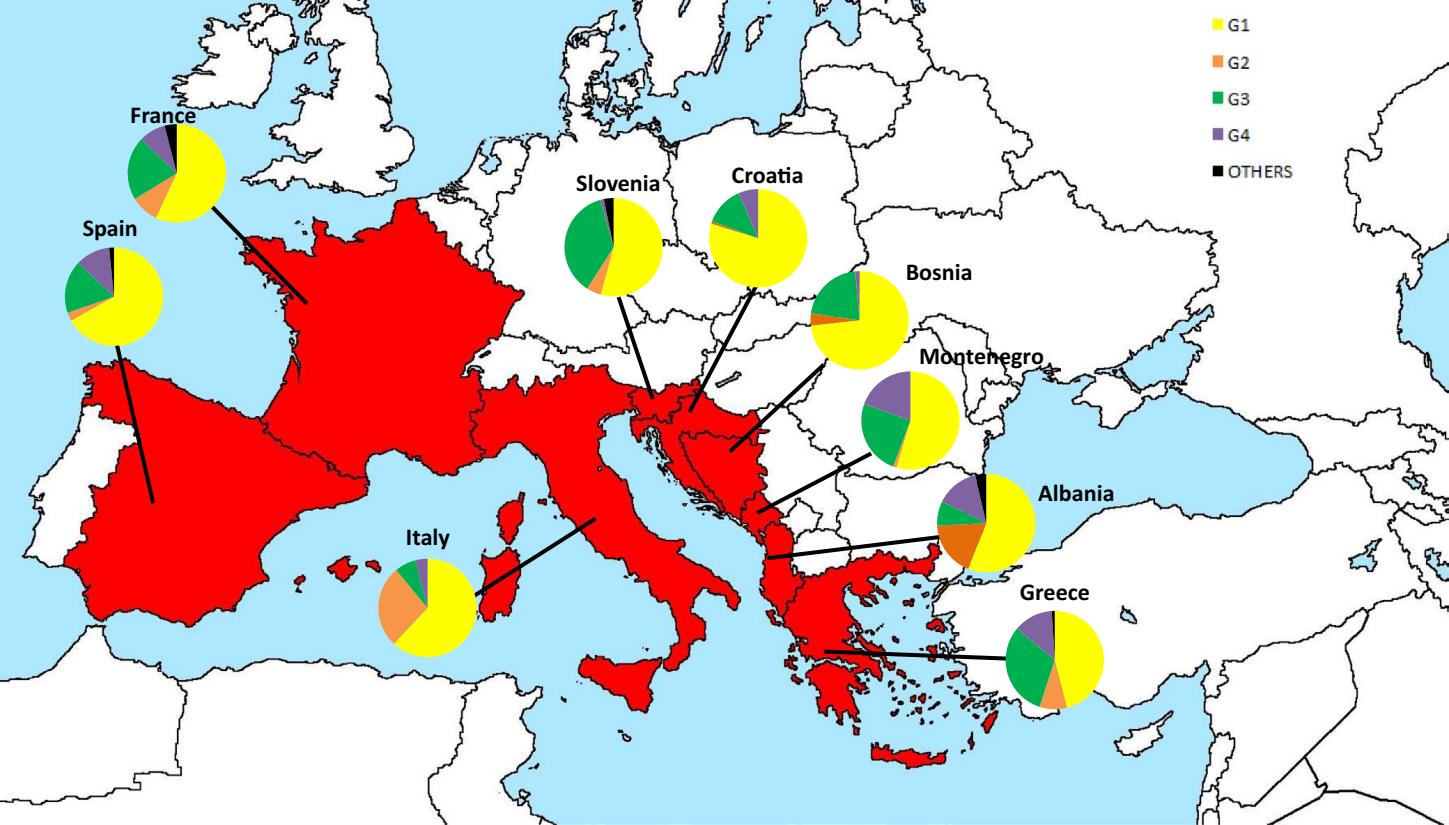
Distribution of hepatitis C virus genotypes in nine European Mediterranean countries

Based on our data, genotype distribution of HCV in the nine selected countries is shown in Table 2 and Figure 2.

**Table 2:** HCV genotypes and subtypes distribution in nine European countries

Countries	Studies (n)	Subjects (n)	Genotypes				
			G1 (%)	G2 (%)	G3 (%)	G4 (%)	Others (%)
Albania	1	50	56	18.2	8	14.2	3.6
Bosnia	1	75	73.3	4	21.3	1.3	0.1
Croazia	6	3541	80	1	12	7	0
Francia	32	13 270	57	9	21	9	4
Grecia	7	2505	46	9	31	13	1
Italia	43	23 041	62	27	7	4	0
Montenegro	2	401	54	1	25	20	0
Slovenia	3	795	56	5	35	1	3
Spain	36	19 407	67	3	17	11	2

Analysis of the distribution of HCV genotypes in Albania is poor and only one article meet the search terms, especially for the limitation of the studied population,^36^ showing G1 as the predominant one with a rate of 56.0% followed by G2, 18.2%, by G4 with a rate of 14.2%, and by G3, 8.0%. Similar is the situation concerning Bosnia, where the only information about HCV genotypes distribution is available from a 2009 study concerning a group of chronic hepatitis C patients reporting the following distribution: G1: 73.3%, G2: 4.0%, G3: 21.3, and G4: 1.3%.^37^

Instead, analyzing the six articles selected from Croatia, we found that G1 is the most common genotype described, (80.0%; 95% CI, 77‐83), followed by G3 (12.0%; 95% CI, 10‐16), G4, (7.0%; 95% CI, 5‐9), and G2 (1.0%; 95% CI, 0.5‐1.5). No “others” genotypes were described.

Regarding HCV genotypes distribution in France, several studies show that G1 is the most common genotype (57.0%; 95% CI, 52‐62), followed by G3 (21.0%; 95% CI, 18‐24), G2, and G4 (both at 9.0%; 95% CI, 8‐10 and 7‐11, respectively). Only a small percentage of “other “genotypes was described (4.0%; 95% CI, 3‐5).

The distribution of HCV genotypes in Greece shows that almost the half of the studied cases are related to G1 (46.0%; 95% CI, 42‐50), followed by G3 (31.0%; 95% CI, 28‐34), G4 (13.0%; 95% CI, 11‐15), and G2 (9.0%; 95% CI, 7‐11).

Also in Italy, G1 is the most frequent genotype (62.0%; 95% CI, 58‐66), followed, however, by G2 (27.0%; 95% CI, 23‐31), G3 (7.0%; 95% CI, 5‐9), and G4 (5.0%; 95% CI, 4‐6), (5‐16). No “others” genotypes were reported.

Regarding the distribution of HCV genotype in Montenegro, G1 is the most common (54.0%; 95% CI, 50‐58), especially among older males, followed by G3 (25.0%; 95% CI, 23‐27), especially among intravenous drug users,^38^ confirming previous data, that however considered Serbia and Montenegro together.^39^ An higher percentage of G4 was described (20.0%; 95% CI, 15‐25).

These data are not very different from those obtained in Slovenia, where G1 is the predominant genotype (56.0%; 95% CI, 53‐59), followed by G3 (35.0%; 95% CI, 32‐38), G2 (5%; 95% CI, 4‐6), “others” genotypes (3.0%; 95% CI, 1‐5), and a small rate of G4 (1.0%, 95% CI, 0.5‐1.0).

Globally the distribution of HCV genotypes in Spain shows that G1 is the predominant genotype, (67.0%; 95% CI, 61‐73), followed by G3 (17.0%; 95% CI, 20‐31), G4 (11.0%; 95% CI, 9‐13), G2 (3.0%; 95% CI, 2‐4), and a very limited rate of “others” genotypes (2.0%; 95% CI, 1‐3).

## 4 DISCUSSION

Hepatitis C virus (HCV) is one of the most common pathogen in Europe, whose epidemiology greatly varies regionally due to the different role of risk factors, adopted screening programs and antiviral treatment rates.^16^, ^40^, ^41^, ^42^, ^43^, ^44^

In this study, we provide a comprehensive review of HCV epidemiology studies throughout nine selected European countries that border the Mediterranean Sea (approximately 200 million of inhabitants) between 2000 and 2017 trying to understand the epidemiological changes in the latest 20 years.

Although this region displays a great heterogeneity especially considering the different historical context and the so variable social status, especially between Southern European countries (Italy, France, Spain, and Greece) and those included in the Balkan area (Albania, Bosnia, Montenegro, and Slovenia), the knowledge of the epidemiological characteristics of HCV infection can give a rational background for better understand not only the recent evolution of the infection in an area so close to Northern Africa and Middle Eastern and consequently strictly related to the migratory fluxes toward Europe but especially for the evaluation of the efficacy of the antiviral therapy, duration of treatment, and future burden of HCV infection.^4^ As widely described, indeed, knowledge of HCV genotypes distribution is an important tool for monitoring the efficacy of therapies whose primary purpose is to eradicate HCV RNA, which is predicted by the achievement of a SVR that seems to be associated with improved clinical outcomes.^45^ G1 and G4 infections are associated with lower response rates and higher treatment duration in response to IFN/RBV combination therapy as compared to G2 and G3.^46^, ^47^, ^48^

Although the introduction of DAAs has solved most of adverse effects of IFN‐based therapy, increasing the rates of SVR the high costs and restricted accessibility of DAA drugs are still the main drivers in the treatment decisions, especially in low‐ and middle‐income countries. Thus, it is clear that in this context, pathogenicity and the duration and cost of treatment are still influenced by different HCV genotypes.^47^, ^49^, ^50^, ^51^

According to our results, G1 is the predominant genotype accounting for 61.2% (95% CI; 53.6‐68.7) of all anti‐HCV infections among adults, ranging from 80.0% in Croatia to 46.0% in Greece. Comparing our data with those collected until march 2016 by The Polaris Observatory, G1 seems to highly increase its prevalence in Croatia (+20.0%) and Italy (+4.0%) while shows a decrease in Slovenia (−14.0%), Spain (−10%), and France (−2.8%).^28^. These data are partially confirmed by ours updated at 2014 (Croatia +21.0%; France −3.0%; Spain −2.0%; Slovenia −2.0%).^24^

G3 is the second most common genotype (19.7%;95% CI, 12.6‐26.7) ranging from 35.0% in Slovenia, 31.0% in Greece and 25.0% in Montenegro to 8.0% and 7.0%, respectively, in Albania and in Italy (Figure 2). Comparing our data with those collected by The Polaris Observatory,^42^ G3 seems to increase its prevalence in Slovenia (+8.0%) and Spain (+9.0%) while shows a decrease in Croatia (−24.0%) and Italy (−3.0%). As previously described, G3 is particularly common among drug abusers especially in West European countries and this may explain its high percentage in the Balkan area.^52^, ^53^, ^54^, ^55^ Matching this data with those collected by 2014, G3 shows a drastic decrease in the studied area (−3.1%) probably related to the introduction of new genotypes as a consequence of migrant fluxes.^24^

G4, instead, frequently associated with Central Africa and the Middle East (5) is the third most common genotype in this area (8.9%; 95% CI, 4.3‐13.4) and ranges from 20% in Montenegro, 14.2% in Albania, 13.0% in Greece, and 11.0% in Spain to the lowest percentages in the Balkan area, with an increase respect to data obtained till 2014 of +1.1%.^24^ Comparing our data with those collected by The Polaris Observatory,^42^ G4 seems to increase its prevalence in Croatia (+4.0%), Greece (+4.0%), and Spain (+1.0%) without any significant changes in the other countries. These data are partially confirmed by ours updated at 2014 (Croatia +4.0%; Greece +6.0%; Spain +3.0%) where we reported also an increase in Italy (+3.0%).^24^ It may be hypothesized that this increase is related to the increasing migrants arrivals in these countries, especially from Middle Eastern countries in war.

G2 is the fourth most frequent genotype (8.5%; 95% CI, 2.5‐15.2) ranging from 27.0% in Italy and 18.0% in Albania to 1.0% in Croatia and 3.0% in Spain. Comparing our data with those collected until by The Polaris Observatory,^42^ G2 seems to increase its prevalence only in Italy (+12.0%) without any significant change in the other studied countries. This genotype is mainly associated with females and mostly detected in older patients. Higher proportions of G2 are typical of Albania^39^ and Italy, especially in Southern areas.^56^, ^57^, ^58^, ^59^, ^60^, ^61^ Although some hypothesis suggest that G2 was probably introduced in Italy as a consequence of Albanian campaign during Second World War,^56^ it is likely that the migration fluxes from Albania to Italy in the 90s may have increased its prevalence in the Southern Italy. Its percentage seems to be stable in the Mediterranean countries in the last 4 years.

Although this review is an attempt for a systematic collection of data concerning the distribution of HCV genotypes in the Mediterranean area of Europe, we cannot deny the existence of a great number of limitations. Firstly, the sample size for some of the countries, especially in the Balkan area, may not produce a reliable estimate, not considering that the use of different genotyping methods in the different studies may affect the obtained results. Then, the heterogeneity among the studies in terms of size of studied populations could make the results more difficult to clarify. Furthermore, a certain proportion of patients in several studies is described as mixed infection, preventing to clarify which was the primary genotype.

In conclusion, the epidemiology of HCV infection shows a high variability across the selected European countries, exhibiting a dynamic process influenced by both the changes of transmission trends and the influence of new migration flows in the last years. Indeed, the 70s and 80s epidemics mainly related to HCV genotype 1 and 2 infections 62, 63 and spread by nosocomial transmission has been partially replaced in the last decades by a new scenario in which genotype 3 and 4 seem to play a more central role. In fact, despite the reduction in HCV transmission by blood products, there is still a considerable rate of transmission related to the drug abusers, especially in the Balkan area. On the other hand, the increasing migration flows, especially from Middle Eastern and Africa, are surely changing the epidemiological scenario of this infection in Europe, introducing new genotypes, like G4, previously not present in this area.

Thus, detailed knowledge of HCV genotypes distribution is important not only from clinical point of view, but it represents a relevant epidemiological tool to monitor the effect of migration flows on HCV scenario in the Mediterranean basin of Europe.

## AUTHOR CONTRIBUTIONS

Loquercio G, Guzzo A, Labonia F, Di Capua L, Sabatino R, Balaban DV, Piccirillo M, Rodrigo L, and Khan NU acquired the data; Petruzziello A. drafted the article and contributed to conception and design; Botti G contributed to critical revision for important intellectual content; all authors approved the final version to be published.
